# Supplementary figures and images for: Reference gene selection for qRT-PCR assays in Stellera chamaejasme subjected to abiotic stresses and hormone treatments based on transcriptome datasets
Source: PeerJ. 2018 Apr 3;6:e4535. doi: 10.7717/peerj.4535 (PMC5888148; doi:10.7717/peerj.4535)

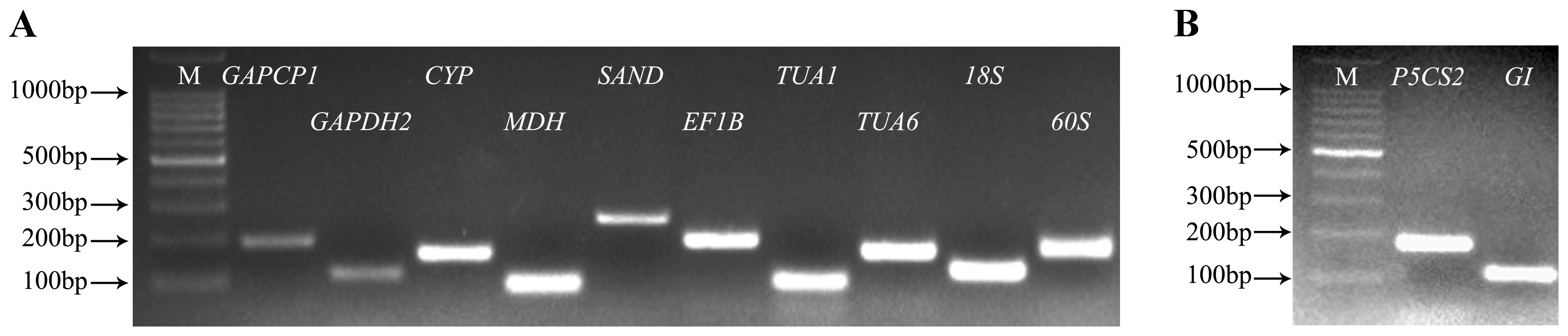

Supplement: Supplemental Information 1 — 2.0% (w/v) agarose low-melt agarose, 1×TAE gel buffer were supplied with 70 V for 1 h. A 100 bp DNA ladder was used to determine approximate sizes of PCR products. The amplified product showed the expected size and no primer dimers. [file peerj-06-4535-s001.png]

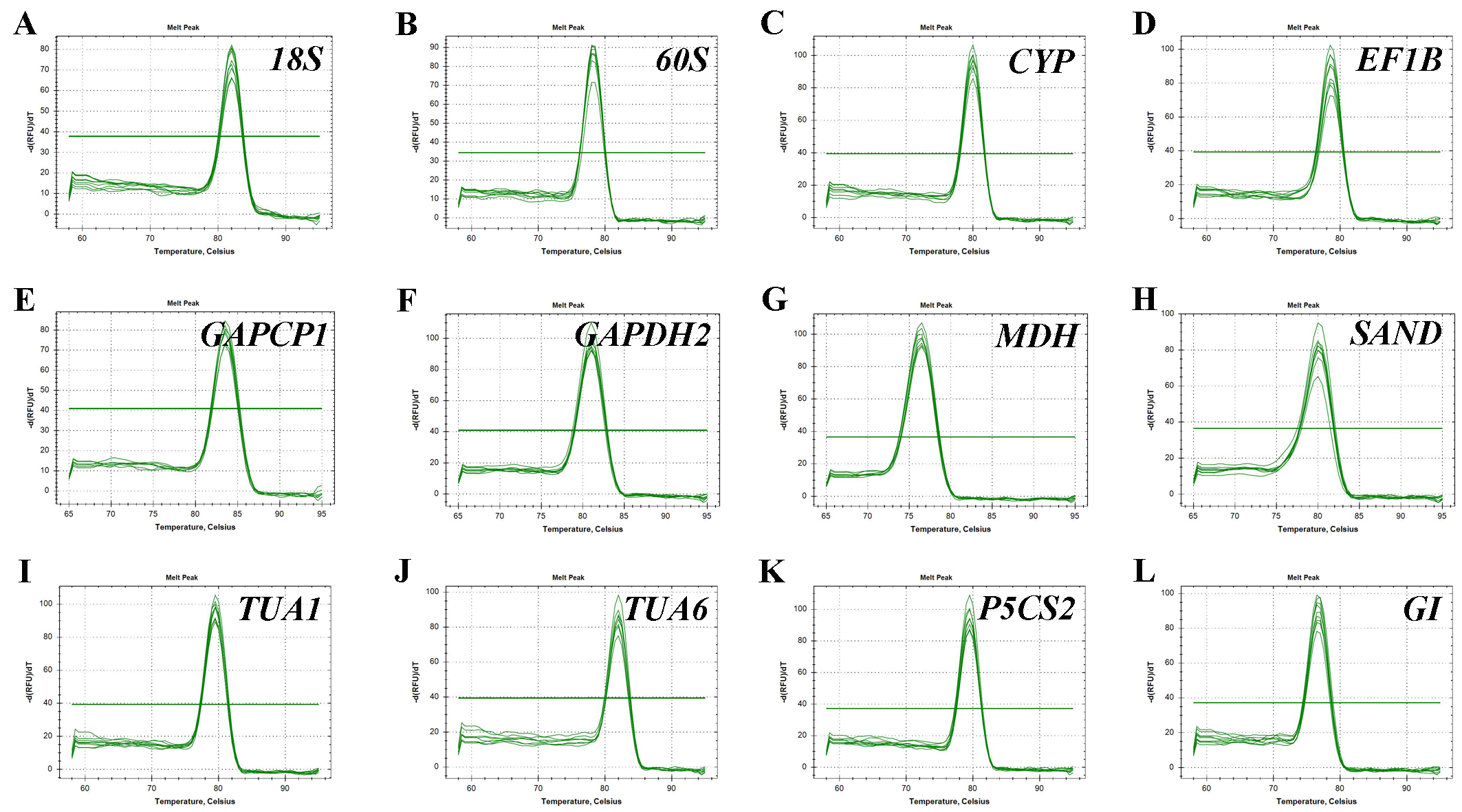

Supplement: Supplemental Information 2 — One single peak was obtained in each amplification reaction. [file peerj-06-4535-s002.png]

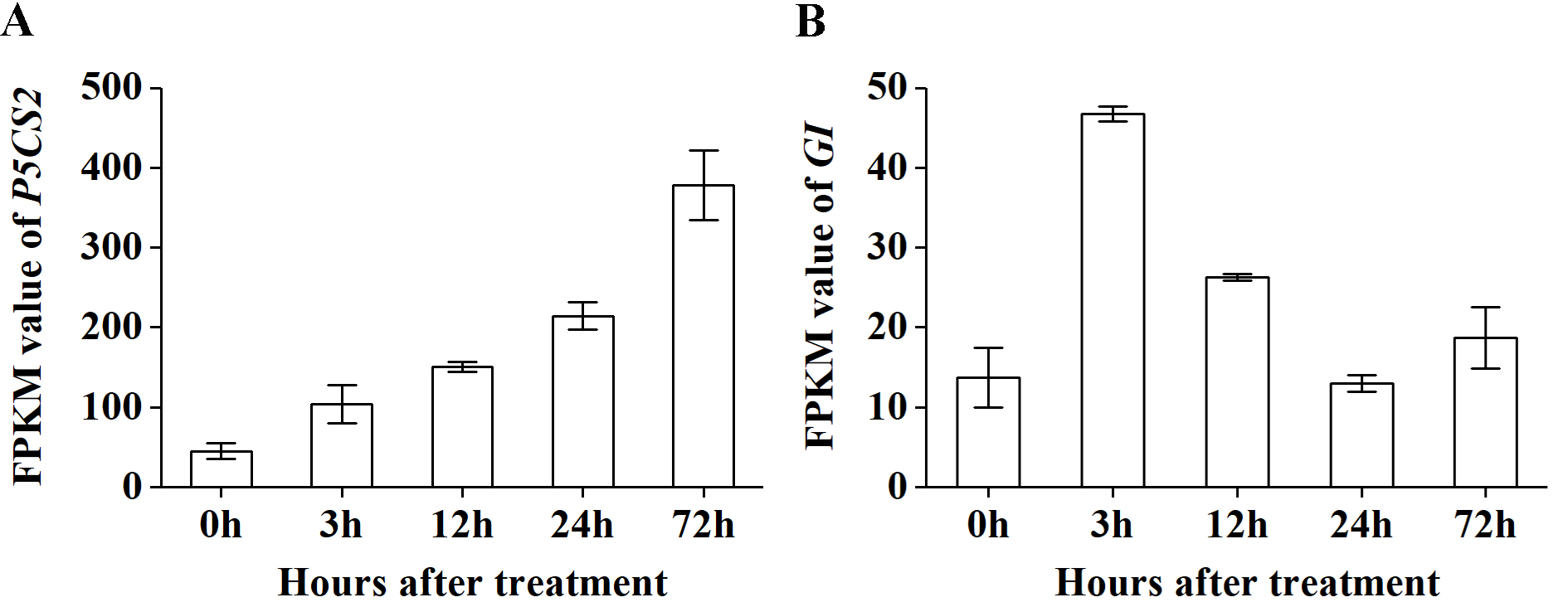

Supplement: Supplemental Information 3 — Bars represent the standard error from three biological replicates. The expression tendencies of P5CS2 and GI from 0 h to 24 h calculated by FPKM value based on transcriptome database were accordance with the results normalized by the most stable RGs combination or only the most reference gene. [file peerj-06-4535-s003.png]
