# Supplementary material for: Reference gene selection for qRT-PCR assays in Stellera chamaejasme subjected to abiotic stresses and hormone treatments based on transcriptome datasets
Source: PeerJ. 2018 Apr 3;6:e4535. doi: 10.7717/peerj.4535 (PMC5888148; doi:10.7717/peerj.4535)
Supplement: Supplemental Information 4 [file peerj-06-4535-s004.docx]

**Sequences of 10 reference genes and two target genes described in this study**

# >c73334.graph_c0, MG516523

GGCTAAATCTCAGCAGATCGTAGTACAAGACTACTCTCATGCTTACAACACCCCGTTCTGTATTTAAGTCGTCTACAAAGGATTTATCTTTCCCAATATTTGAAATTACAATTCAGCATACTCTCCACTACCAACACTTGTCAGGCAATGTGAGTGCGTCGCAATTTTATGATGTTGGATGCCCAAAGGACATCTATCGCTATTCGCGGTACATCAGGAAAGGTGATTATCGTCTATTCCAGCATGGATTCTAACTTAGAGGCGTTCAGCTATAATCCTACAGATGGTAGCTTGGCAGCACTAGCTTTTCAGCTAACTGCAGTTACCAATTATCTGAACCAACGGTTCCTCTCGTACTGGGTTGGATTACTGTTTAGACAACGAACTCATCAGTAGGGTAAAACTAACCTGTCTCACGACGGTCTAAACCCAGCTCACGTTCCCTATTGGTGGGTGAACAATCCAACACTTGGTGAATTCTGCTTCACAATGATAGGAAGAGCCGACATCGAAGGATCAAAAAGCAACGTCGCTATGAACGCTTGGCTGCCACAAGCCAGTTATCCCTGTGGTAACTTTTCTGACACCTCTAGCTTCAAATTCCGAAGGTCTAAAGGATCGATAGGCCACGCTTTCACGGTTCGTATTCGTACTGAAAATCAGAATCAAACGAGCTTTTACCCTTTTGTTCCACACGAGATTTCTGTTCTCGTTGAGCTCATCTTAGGACACCTGCGTTATCTTTTAACAGATGTGCCGCCCCAGCCAAACTCCCCACCTGACAATGTCTTCCGCCCGGATCGACCTGCCGAAGCAAGCCTTGGGTCCAAAAAGAGGGGCATTGCCCCGCTTCCGATTCACGGAATAAGTAAAATAACGTTAAAAGTAGTGGTATTTCACTTGCGCCCGAGGGCTCCCACTTATCCTACACCTCTCAAGTCATTTCACAAAGTCGGACTAGAGTCAAGCTCAACAGGGTCTTCTTTCCCCGCTGATTCTGCCAAGCCCGTTCCCTTGGCTGTGGTTTCGCTGGATAGTAGACAGGGACAGTGGGAATCTCGTTAATCCATTCATGCGCGTCACTAATTAGATGACGAGGCATTTGGCTACCTTAAGAGAGTCATAGTTACTCCCGCCGTTTACCCGCGCTTGGTTGAATTTCTTCACTTTGACATTCAGAGCACTGGGCAGAAATCACATTGCGTGAGCATCCGCAGGGACCATCGCAATGCTTTGTTTTAATTAAACAGTCGGATTCCCCTTGTCCGTACCAGTTCTGAGTTGGCTGTTCGACGCCCGGGGAAGGCCCCCGAAGAGGCCGTTCCCAGTCCGTCCCCCAGCCGGCACGCAGCGACCCGCTCTCGCCGCGGTAGCAGCTCGAGCAGTCCGCCGACAGCCGACGGGTTCGGGACTGGGACCCCCGTGCCCAGCCCTCAGAGCCAATCCTTTTCCCGAAGTTACGGATCCATTTTGCCGACTTCCCTTGCCTACATTGTTCCATCGACCAGAGGCTGTTCACCTTGGAGACCTGATGCGGTTATGAGTACGACCGGGCGCGAACGACACTCGGTCCTCCGGATTTTCAAGGGCCGCCGGGGGCGCACCGGACGTCGCGAGACGTGCGACGCTCTTCCGGCCGCTGGACCCTACCTCCGGCTGAGCCGATTCCAGGGTGGGCAGGCCGTTAAACAGAAAAGATAACTCTTCCCGGGGCCCCCGCCGACGTCTCCGGACTCCCTAACGTTGCCGTCAGCCGCCGCGTCCCGGTTCGGGAATTTTAACCCGATTCCCTTTCGAAGCTCGCGCCAAAGCGCTATCGGACGGGCTTCCCCCGTCTCTTAGGATCGACTAACCCATGTGCAAGTGCCGTTCACATGGAACCTTTCCCCTCTTCGGCCTTCAAAGTTCTCATTTGAATATTTGCTACTACCACCAAGATCTGCACCGACGGCCGCTCCGCCCGAGCTCGCGCCCTGGGTTTTACAGCGACCGCCGCGCCCTCCTACTCATCGGGGCCTGGATCTTGCCCCGACGGCCGGGTGTGGGTCGCGCGCTTCAGCGCCATCCATTTTCGGGGCTAGTTGATTCGGCAGGTGAGTTGTTACACACTCCTTAGCGGATTTCGACTTCCATGACCACCGTCCTGCTGTCTTAATCGACCAACACCCTTTGTGGGATCTAGGTTAGCGCGCAGTTGGGCACCGTAACCCGGCTTCCGGTTCATCCCGCATCGCCAGTTCTGCTTACCAAAAATGGCCCACTTAGAGCTCTCGATTCCGTGGCACGGCTCAACAAAGCAGCCATGCCGTCCTACCTATTTAAAGTTTGAGAATAGGTCGAGGGCGTTGCGCCCCCGATGCCTCTAATCATTGGCTTTACCCGATAGAACTCGCACGAGCTCCAGCTATCCTGAGGGAAACTTCGGAGGGAACCAGCTACTAGACGGTTCGATTAGTCTTTCGCCCCTATACCCAAGTCAGACGAACGATTTGCACGTCAGTATCGCTGCGGGCCTCCACCAGAGTTTCCTCTGGCTTCGCCCCGCTCAGGCATAGTTCACCATCTTTCGGGTCCCGACAGGCATGCTCTCACTCGAACCCTTCTCAGAAGATCAAGGTCGGTCGGCGGTGCACCCAAAGGGATCCCACCAATCAGCTTCCTTGCGCCTTACGGGTTTTCTTGCCCGTTGACTTGCACGCATGTCAGACTCCTTGGTCCGTGTTTCAAGACGGGTCGAATGGGGAGCCCGCAGGCCGACGCCAGGAGCACGCAGATGCCAAGGCACGCCAAGAGGCGCGTGCTGTAACCCACAATCATGGCGACGTCATCTCCATGCGCAAAACAAAAGCACAGGCTTAGGACGCCACCACAATCCGCATCGGTCCACATCCCGAGACGATCGACGGACCGGTTTTCACCGTTCCGCATCCGACCGGGATGCATCGCCAGCCCCCATCCGCTTCCCTCCCGACAATTTCAAGCACTCTTTGACTCTCTTTTCAAAGTCCTTTTCATCTTTCCCTCGCGGTACTTGTTCGCTATCGGTCTCTCGCCCGTATTTAGCCTTGGACGGAATTTACCGCCCGATTAGGGCTGCATTCCCAAACAACCCGACTCGTAGACAGCGCCTCGTGATGCGACAGGGTCCGGGCACGACGGGGCTCTCACCCTCTCTGGCGCCCCTTTCCAGGGGACTTGGGCCCGGTCCGTCGCTGAGGACGCTTCTCCAGACTACAATTCGAACGCCGAGGGCGCCCGATTTTCAAGCTGGGCTATTCCCGGTTCGCTCGCCGTTACTAGGGGAATCCTGGTAAGTTTCTTTTCCTCCGCTTATTGATATGCTTAAACTCAGCGGGTATTCCCGCCTGACCTGGGGTCGCGATGTGCATTATCTTTGAATGAAGGAATTCAAAGTACAAAGACAACCAACTCTAATGACATGCTGCACACGATGACTTAACGAGGGCAAGTCTAAACAAACCACCGCTCATCATGGCATACACCACATTAGGGTTACTATATTTAGGCCAACCGCGCTACCATGAGGACGGGAGAGCCATGATCAGCCTCAAACCAACATAGGTGGTAGGGGCTACGATGCGTGACACCCAGGCAGACGTGCCCTCGGCCCGAAGGCTTAGGGCGCAACTTGCGTTCAAAGACTCGATGGTTCACGGGATTCTGCAATTCACACCAAGTATCGCATTTCGCTACGTTCTTCATCGATGCGAGAGCCGAGATATCCGTTGCCGAGAGTCGTTCTTTATAATATACATATCACGCATGACCCCACACAATCTTGTGATGGCATGGGGCAAACGTTTCACTTATCACAATTCCTTGGCGCAATCCGCGCCGGGGTTGGTTATCTTGGCCACATGGGCCAGTTAAGAGGAAATGGAGCCAATGACACCATGACCCCTCAACACATTTGCTAACATGTTCACGAGTTGTTCTGCTAAGCAGGATTCGACAATGATCCTTCCGCAGGTTCACCTACGGAAACCTTGTTACGACTTCTCCTTCCTCTAAATGATAAGGTTCAGTGGACTTCTCGCGACGTCGCGGGCAGCGGACCGCCCTCGTCGCCGCGATCCGAACACTTCACCGGACCATTCAATCGGTAGGAGCGACGGGCGGTGTGTACAAAGGGCAGGGACGTAGTCAACGCGAGCTGATGACTCGCGCTTACTAGGAATTCCTCGTTGAAGACCAACAATTGCAATGATCTATCCCCATCACGATGAAATTTCAAAGGTTACCCGGGCCTGTCGGCCAAGGCTATAAACTCGTTGAATACATCAGTGTAGCGCGCGTGCGGCCCAGAACATCTAAGGGCATCACAGACCTGTTATTGCCTCAAACTTCCGTGGCCTAAGCGGCCATAGTCCCTCTAAGAAGCTAGCCACGAAGTATACTCCGTGTAGCTAGTTAGCAGGCTGAGGTCTCGTTCGTTAACGGAATTAACCAGACAAATCGCTCCACCAACTAAGAACGGCCATGCACCACCACCCATAGAATCAAGAAAGAGCTCTCAGTCTGTCAATCCTTACTATGTCTGGACCTGGTAAGTTTCCCCGTGTTGAGTCAAATTAAGCCGCAGGCTCCACTCCTGGTGGTGCCCTTCCGTCAATTCCTTTAAGTTTCAGCCTTGCGACCATACTCCCCCCGGAACCCAAAAACTTTGATTTCTCATAAGGTGCCGGCGGAGTCCTTAAAGCAACATCCGCCGATCCCTGGTCGGCATCGTTTATGGTTGAGACTAGGACGGTATCTGATCGTCTTCGAGCCCCCAACTTTCGTTCTTGATTAATGAAAACATCCTTGGCAAATGCTTTCGCAGTTGTTCGTCTTTCATAAATCCAAGAATTTCACCTCTGACTATGAAATACGAATGCCCCCGACTGTCCCTGTTAATCATTACTCCGATCCCGAAGGCCAACACAATAGGATCGGAATCCTATGATGTTATCCCATGCTAATGTATCCAGAGCGTAGGCTTGCTTTGAGCACTCTAATTTCTTCAAAGTAACAGCGCCGGAGGCACGACCCGGCCAGTTAAGGCCAGGAGCGCATCGCCGGCAAGAGGGACGAGGCAACCGATGCTCACTAAAAGCGGACCGATCGACCCACCCCAAGGCTCAACTACGAGCTTTTTAACTGCAACAACTTAAATATACGCTATTGGAGCTGGAATTACCGCGGCTGCTGGCACCAGACTTGCCCTCCAATGGATCCTCGTTAAGGGATTTAGATTGTACTCATTCCAATTACCAGACTCTAAGAGCCCGGTATTGTTATTTATTGTCACTACCTCCCCGTGTCAGGATTGGGTAATTTGCGCGCCTGCTGCCTTCCTTGGATGTGGTAGCCGTTTCTCAGGCTCCCTCTCCGGAATCGAACCCTAATTCTCCGTCACCCGTCACCACCATGGTAGGCCTCTATCCTACCATCGAAAGTTGATAGGGCAGAAATTTGAATGATGCGTCGCCGGCACAAAGGCCATGCGATCCGTCGAGTTATCATGAATCATCAGAGCAACGAGCGTGAGCTCGCGTCGACCTTTTATCTAATAAATGCATCCCTTCCAGAAGTCGGGGTTTGGTGCACGTATTAGCTCTAGAATTACTACGGTTATCCGAGTAGCAAATACCATCAAACAAACTATAACTGATTTAATGAGCCATTCGCAGTTTCACAGTCTGAATTTGTTCATACTTACACATGCATGGCTTAATCTTTGAGACAAGCATATGACTACTGGCAGGATCAACCAGGTAGCATTCCTCATTCGATGTCAACCTTACACACATAATAAACATGCATGAGCATGAATAGTACGACGCAAGGGAGAGCATCGATCAAAAGGAGGTGTAATGCCTCGGGACAGGTTAATAAGTCGGTCAACCCACATATCAACCACAATATTTTCCACATCCTAGCTCACAATCAACATCCATGAGTGCCTAGGAGCCACCACAGAGTGGAACTCGTGGACCTCATGGAGCGATTTTTAGTTGCCCATAGACCACTTTAATGAGTCACGGGACAAATGCATTGAACGCCACAACAATATACCTTGTTATGTTAGGTACGCAGCACAGGATCTCATCATCCCCCAAGCAAAAATATTGCTAGGAGTGCAATTGAAGAGGATGCTTTGTTAGTAGTTCGGTGCACAAGCACAGAGCCTACCAACACAAACATCCTTATAACCACTCACACACCCATAGCGAAAGCATGGCAACAACCTCACAAGGGCACCAACACACCTTGACCCCAATTGGACCAAGGGATGCAAGCAAACCAAATAAGGTCATCAACATCATCGATAGGCATGAGCTAAAAGTTTTGTTAAGAAAACAACCGATGTCAACCACCCGAATCACCACAAATGGCAACGCCAGCAACAAGTACCAACGAAGTCTCCATTTGTCGGAAATACCACAACCCTCATGCGACTTCGCTAACCCGCAAATATCTCCATTTGTCGGGTCAAACCGCTATCCAGGGGAACCGAAGCTTATCCGAAAAAAGCAATGCCAGCAACAAGTACCTACAAACACTCAATTTTCGGAAGCTGGACAACAATCAGCGCGAGCCAAAAGTTGGACAGAGCTAGCGGTGGCCGGAATAGCGGTGCGTCGCTTGAGGGACGGTGGTGGGTCATTTTCCGACAAGGGACCCCGAGGCAACATCAATACTCACCCACGAGCTAGCAAGAAGGTCTCACACACATGCGGCAGCCTCAGCTACCGCATGTATGTGAGACCCCCGAGGGGGCACAAGGCTAGCATCACCCCCCTAAAGAGCATTATTGACTTTAGGAGGATTCCTGGAAGGAAACATCAAAAACTTGAGATGACACCCTATCCATAAAACGATGTAGAAATGTTATATTGATGCCATTTTTTGAGGGAAACTTGAAATAAATACAATCTAACTAGTTCCAAAATTTCAGAAATTTTTGACGACTAAATAATTTAATATTAATTTATTATGCTTTTTATCATTAAAAAATCATAAAAAATAGGAAAATGGTCATATGAGTCTCAAACTTTGCAAACCCTCATCATAAATCGTGACTCAAATTATGGAATCATTTTTGGATGAAAGGCTTGAAAAGTTGTCGACAAAGTTGAAGTCATTGCACTATGAGGTGCTCCCGGGGTGCCCTCGCAAGCCCCAATATGTGTTGTTCTACACAAAACTTGCAAGACTCCCCCAATAATCCCTACCAATCATGCATCCATTCCCTTCGCAATATCATATAGAGATGGGAGGAGCTCATGGGACTCCGAAAACTTGAGAATTCCTAAG

# >c68075.graph_c0, MG516524

CCACGAGCCAGCCTCCCAACTCTCTAGGGTTTCTTCTCTTCCTCTACAACAAAACCGTCCACCTTCCCGGAGGTTAAAGCGACAGCGCCGCCCTGCTGTCCGCCTTGGCTGAGCTCCAGCGTCGGTCTTGGCCCTCGGTTTTGCTCTGTAGTGCAGTTAGTTCTCTCATATCTTACTTTTGTAGATTTTTTCCGTACTCGAAAATGCCGTCCAAACTATCCAAGGCCGACAAGAAGATCGCTTACGATGGGAAGCTCTGCCAGTTGCTTGACGAGTATACCCAGATCTTGATCGTGGCGGCCGACAATGTGGGCTCGAACCAGCTCCAGAACATCCGGAGGGGTCTTCGTGGCGACTCTGTTGTGCTTATGGGCAAAAACACCATGATGAAGCGCTCGGTTAGGATTCATTCTGAAAAGACTGGCAACAAGGCTTTCCTCAACTTGATCCCTCTCCTTCAGGGAAATGTGGGTTTGATTTTCACCAAGGGTGATCTGAAGGAAGTCAGCGAGGAGGTTGCTAAGTACAAGGTTGGAGCTCCTGCTCGTGTTGGGTTGGTTGCACCAATTGATGTGGTTGTCCCACCTGGAAACACTGGGCTCGATCCATCTCAGACATCTTTCTTCCAGGTGCTCAACATTCCAACCAAGATTAACAAGGGTACTGTTGAAATCATTACCCCTGTCGAGCTCATCAAGAAGGGTGACAAGGTCGGGTCTTCTGAAGCTGCGTTGCTTGCGAAGCTTGGAATTAGGCCCTTCTCATATGGTTTGGTTGTTCTGTCTGTTTATGACAATGGATCTGTGTTCAGCCCAGAGGTTCTAGACTTGACTGAGGATGACCTCCTGGAGAAGTTTGCTGGTGGTGTTTCCATGGTCACATCCTTGGCTCTTGCCATCTCATTCCCAACCCTTGCAGCTGCACCACACATGTTCATCAATGCGTACAAGAATGTCCTAGCAGTTGCCGTTGCTACTGAGTACACCTTCCCTCAAGCTGAGAAAGTTAAGGAGTTCTTGAAGGACCCAAGCAAATTCGCTGTTGCGGTGGCTGCTGCTGCTCCAGCTGCTGGTGGTGGTGATGCTCCAGCTGCAGCTTCCAAGGAGGAGGAAAAGAAAGAGGAGCCTGCTGAGGAGTCTGATGAGGATATGGGCATCGGGTTGTTCGACTAGTTGTTCGATAGCATCCGTCTCTCATACAAGTTGTGTTCTTTTCATCTGTTTCGACAGTTTGTGAACTGATTGGAAGATTTGTTCCGCTGTGTACTCTATGTTCCTTAGGACTTGATCATGTTTTGTTCCTATAGAGGATTGTGCGAGTTGCTTCCGTTGTTTGCTTTTATAGGCCCGGCCGCCGTTAATGCATACTCTATGTTCCTTAAGATGGTTAAAAAGCATTTATTTTTAATTTGTTCAAGCG

# >c71629.graph_c0, MG516525

TCATCAAAATTCAGGGTACTAATTTGGCCATAAAACTACTCTATTCTATACGAGTACATGATCAATACAATAGAGGCAATAGCAGGCGGGGCCTAGATGCTAATTGGAGAGCCCAAAAAGCACTGGGCCGAATCGGCCCAATCAATCCTGTTTCCTTTGTCGGACGACTTTACAGATAAGGTAGTTGGCAATGGCGGAAGCAGAGGCAGACAGTTCCATATCCATCTGCGATTGCCCTCGCCTCGCCTCCATTGAAATCTAGCAAAGCAAAGCAATTACAGTACAATACAATGCTTTCTCTCACTTCAGCCTCTCTAAAACCCAAAACCCTCTTCTCTTCTCCCTCCTCCGCCTCCACTTCCAAATCACTCCTTAAACCCATCTCCGCCATCATTATCCCCTCCAGTTCCAAGCAGCCGCCGCCTCAGCAGCAGCTTTACCAGCCCTTCCGCCCACCGCCATCGCCTCTCCCCTCCAAGTTCCGCTCCCTCGATGCCATCGGCCGCATCGACGTTCTCGCCAACCGCCTCGGCCTCTGGTTTGAGTACGCTCCTCTCATCTCCTCTCTGTACCAGGAGGGATTCACCCCTCAAACCATCGAAGAAGCTACCGGAATCACCGGCGGTGAGCAGAACCGCCTGATCGTCGGCGCCCAGGTGCGCGAGTCGCTCATCCAGTCCAACACCGACCCGGACGTCATGGGCTTCTTCGACACCGGCGGCGCTGAGCTTCTGTACGAGATACGCCTTCTGAGCGCCTCGCAGCGGGCTGAGTCCGCCCGATTCATCGTGGAGAATTCCTTGGATATGAAAGGTGCTGAGGATTTGGCTCGGTCGATAAAAGATTTCCCTCGTCGCCGTGGAGACAAGGCCTGGGATCAGTTCGATTACAAATTACCAGGAGATTGTCTGGCGTTTATGTATTTCAGGCAGAGTAAAGAACATAGGAATCCTTCGGTGCAGAGGAATGCTGCTCTGCAACAGGCTTTACAAGTGGCCGGTTCAGAGAGTGCTAAAAGTGCTATTCTGGCGGAGATTGAGGGTGGAGAGGCCGAGGAGGAGGAAGAGAAAATCGATGATATTCGAGTTCCGGTGGTGAGGTTGAAGTTTGGGGAGGTGGCAACGGCGAGCAATGTGGTTGTTTTGCCAGTTTGCAGGGCTGAGGAGGTTTTGGAGGCACCATGGGATTGTAAGAGGGAGGGGGCATTCGGTGTGGTGGTGGCGGAGAAAGGGTGGCAGAGGTGGGTTGTTTTGCCGCAATGGGAGCCCTTGGCTGCTTTGGCTAAAGGCGGAGGCGTGGTTGTGTCATTTCCTGATGCAAGAGCATTGCCATGGAGGGTGAATAGGTGGTACAAGGAGGAGCCAGTTTTGGTGGTGGCCGATAGAGGAAGGCAGACTGTTGAGGTGGATGATGGGTTCTATTTGGTGGCCACCGGCCATGGTGCTGATCTCAAGGTGAGGCGGGGCTCGGAGTTGAAGGTGGAAGGAGTGGAGGAGAGTTTAGGAACCGTGATTGTGGTTGTGAGGCCACCCAAGTACGAGACTGATGATCAGTTAAGTGATGAAGATTGGGAATGAAGAAAATAGCTGCAATGGGAGCAACTTGAGTAATTTTTTTGACTCCACTTTAGACTCTTAGTCTTCAAAATTAGTCATTATGGAAGATCTTCTTGTGGACAAGTTTGTATTGCCTGCAGAAGCAGCTCAATTTTTGTTTCACATGTACCAGTAATACACTCAACCTATTCATCTTACACAAGTTCATCTTTGCAACATTATAATACAAGCCATTTGAATGATAAATGATAATGCCACAATTAACTTGATACATAGTTTGAGGCAACCTAGCAGTTGTGCAGCAGGAAACAGATGAACTGTAAGAGACTAGAAGGAGACGGTCACTACCTGTGTGTGATTCCTTCTTCGCAACTATCAATTCTCTGATAACTGTCCACAGTCTTCTATAACGACTGTCTGCGAAGGTGTACCATGTTCAGAACCTACCTTTTCCATCTGCTGAACAACATTGTAACCATCCACAACCTTACCAAACACCACATGCTTCAAATCAAGCCACTTTGTCTGCGCTGTGCATATGAAGAACTGAGACCCGTTTGTATCTGGTCCAGCATTTGCCATTGACAGACACCCAGGGCCACTGTGCTTCAGCTTGAAGTTCTCATCTGCAAACTTCTCACCATAAATGGATTCTCCTCCAGTACCATTCCCTTTTGTGAAGTCACCACCTTGGCACATGAAACCCGGGATGATACGGTGAAATGCCGAGCCTTTGTAATGCAATGGTTTCCCCGCCCTTCCAATTCCTTTCTCACCAGTGCACAGGGCACGGAAGTTTTCAGCAGTCTTTGGCACTGCATCAGCAAACAACTCCATTACAACTCTACCAGCTTTTGCTCTCCCAATTGCTATGTCAAAGAAAACTTTTGGATTGGCCATTCTTCTTCAACGTGTTCTCCAAAAAACTCAAGTCGACGAGGCAAAGACCACAGAGTTCGATAACCAACCTAAGGGTATTAG

# >c67520.graph_c0, MG516527

TGAGTATATTATATTGGTCTCCTCCACCTCCTCCTCCTACTCCTCCGCTGCTTGCCATCTCCCAAAAGCCTAGCCTTCTTCTCGTCTCTTCTCTAGGGTTTTTGCGACCGAGGCCCTCTCCTCCCTCCCATGGCTTCTTCTTCTTCGTCTTCAGCTCATGCCATTAGATCCGTCGCCTGTTTCCGTTCGGACCTATCGCCGCCGTCCTTCAAGGCGTCGTCGAGCCTTGGTTTCAGCCGTAATGTCAACTTCTCCAAGACTCCCTCAACTTCGCTCTCTTCACCTTTCCAGCAATGCAGTTTCAGAAGTTTTCAACCCATCAGAGCCACAGCTACAGAAGTGCCACCAACAATTCAGAAACCACAAACAAGCGGGAAGACAAGAATCGGAATCAATGGTTTCGGCCGCATTGGAAGGCTGGTTTTACGTGTTGCAACATCAAGGGATGATATTGATGTCGTGGCAGTCAATGATCCTTTCATTGATGCCAAGTACATGGCTTACATGTTTAAGTATGATTCAACGCATGGAGTCTTCAATGGGACTCTCAGGGTTCTGGATGAATCCACCTTGGAAATCAACGGGAAGCAAATTAAAGTTGTGAGCAAAAGAGACCCAGCAGAGATTCCATGGGGTGATTTAGGAGCCGAGTTTGTGGTCGAATCATCTGGTGTTTTCACAACATTGGATAAGGCCTCAGCACATATCAAGGGTGGTGCAAAGAAGGTTGTTATTTCAGCTCCTTCTGCTGATGCACCGATGTTTGTGGTAGGAGTAAATGAGCAGTCTTATAAACCAAATATGGACATCGTTTCTAATGCAAGCTGCACTACAAATTGTCTTGCTCCTCTAGCCAAGGTGGTTCATGAGGAATTTGGCATTATTGAGGGGTTGATGACAACTGTGCATGCAACTACAGCCACACAGAAGACTGTTGATGGTCCTTCAATGAAAGATTGGCGAGGTGGGCGTGGAGCTGGACAAAATATAATCCCTAGCTCTACTGGTGCTGCAAAGGCTGTTGGCAAAGTCCTTCCTGAACTTAATGGAAAGCTTACTGGAATGGCATTTCGAGTACCCACACCTAACGTTTCTGTTGTTGATTTAACTTGTCGACTTGAGAAGAGTGCATCTTATGAAGATGTCAAAGCTGCAATCAAGTATGCTGCAGAGGGGCCACTTATGGGCATTCTTGGGTATACGGATGAGGATGTTGTCTCTAATGACTTTGTTGGGGACTCGAGGTTAAAAATTTCTCTCCCTTGCCTCTTTGGCATGCACATTCTTAAACATGCACGTTTCTTAATTGAGATGAACTGTAATATCTGGTGCTCACAGCTTGTGTGAATGTTGTCAACCCACCTTGCAAGGATCAAGGTTGAGGGCTTTGACCTATCATGAGCCTGCCATCCCCTTCTTTTTAATATGAGGGAGTGCGAGGATTTATCTTTTCTTTTTAATATGCTCTGATTGAGTGCCATACTCGCCATGAAATGGTGGATATGCTGTTTGCAATTTAGAAATTTTCTTGGACAAAACCAAGAGAACCTTAGGCTAAGATGTTTGAGAGAAAGATGTCAGATGATAATTTGTTTCCAGAGTTTAGTCGGTGTGATTTGATCTGACACTTTCTTGTCTCATCAGGTCAAGCATATTTGATGCGAAGGCTGGAATTGGGTTGAATAACTCCCTCATGAAACTCGTGTCCTGGTATGACAATGAATGGGGC

# >c74212.graph_c0, MG516528

ATTAAATACCTTCCACTTGCACTCTTCTTCTTCCCAAAAAGCTGCTCTCATCTTTCTATCCAAACCTTGCTGCTGCTCGTTATCATTGCCAATCGCCATGGCTTCGCAGAAGAAGATCAAGATCGGAATCAATGGTTTCGGAAGAATCGGTCGCTTGGTTGCCAGAGTTGCTCTGCAGAGGGACGATGTTGAGCTCGTCGCTGTCAACGATCCCTTCATCACCACTGATTACATGACCTACATGTTCAAGTACGACAGCGTCCACGGCCAGTGGAAGCACCACGAATTGAAGGTCAAGGACTCTAAGACTCTTCTCTTTGGTGAGAAGCCGGTCGCCGTTTTCGGATTCCGCAACCCAGAAGAGATTCCTTGGGGTGAGACAGGTGCTGATTTCGTTGTTGAATCTACTGGAGTTTTCACTGACAAGGATAAGGCAGCTGCTCACTTGAAGGGTGGTGCTAAGAAGGTTGTCATCTCAGCACCCAGCAAGGATGCTCCCATGTTTGTTGTGGGTGTCAATGAGAAGGAATACAAGCCTGAATTGGACATTGTTTCCAATGCTAGCTGCACCACCAACTGTCTTGCTCCATTGGCCAAGGTTATTAATGATAAGTTTGGAATTGTCGAGGGTCTCATGACCACTGTCCACTCAATCACTGCCACACAGAAGACAGTAGATGGACCTTCAGCAAAGGATTGGAGAGGTGGTAGAGCTGCTTCCTTCAACATCATCCCTAGCAGCACTGGCGCTGCCAAGGCTGTTGGAAAAGTTCTGCCTTCTTTGAATGGCAAGTTGACTGGAATGTCTTTCCGTGTTCCGACTGTGGATGTCTCAGTTGTTGACCTCACTGTCAGGCTTGAGAAGGCAGCATCTTATGATGACATTAAAGCTGCCATCAAGGAAGCATCCGAAGGCGATCTCAAGGGAATTATGGGTTACACCGATGAGGATGTTGTGTCTACCGACTTTGTTGGGGATAGCAGGTCAAGCATCTTCGATGCCAAGGCTGGCATTGCTCTAAGTCCCAACTTTGTGAAACTGGTCTCCTGGTATGACAATGAATGGGGTTACAGTTCTCGTGTGATCGACTTGATTGTCCACATGGCCTCTTGCTAGTCTGCATCATGCTATAAGCGTTGCCTGGGTTGGTTTCAAGGATTTCATGCTGGTTTAGTGTTTGAGGCTTAGACATGGTTACAGTACCAGAGTTACTGGATTGAAAATAAAAGCTTGTGGATTGCTTGTTTTGCAAGTTTTCTTATGTTTTCCATTTCATTTTTGGAGTAGTCAGTTCAGAGGGTTTATAGCTTTCCGAACTTTTTACCATTTTCAATTTCAGAAATAGAGATTGTGGTCATATTGCAGTTACTGTCCTTCCCAGTGTTATCAAATTGTGATTATTTTGTCACACTTCTCATTTCGGAAATCAATTTAGTTTAGTAATGCAAATCAATTTGGTTCTAGAAAATAAGAAGGAAATGTCTTCAAGACTTCAAGAAAACAACTTTCAGTGATTAATTTGGTTATAAATGGGCATATGTACGGCTGGCTATTTACAACCAAAAGCCAATCCTTCTAATTACAACCTTACCCATTTAAGAACATGAAGTACAAGCTCAAGGGTGGCTTCAAAGGGAAAGGGTCCCTTGTCCAGTACAACACCTGAACCCTGTGATTCTCAAGGGGTGACCCGGGAAAATGCAACAGAAGGACACAACGCCCCCGATCCAAGATTTTTGTTCCAATGAAATACTCCCTCTCTGTCCCAAAGTACTATCAACACATCAATAAAAAAAAAGTTTAAAATGAAATGATATTTTTATATTATTTTAGTCCACTTTTACCATTGGAAATGCAC

# >c70711.graph_c1, MG516529

CAAAGAAGTGGGGGGTGGCGGAATGCTAGTGGGCCCTAAACTTGAGATCATGGACGGGAGGTAAGCTTGTCTTGTCCAATAAAGCAGAAAGCGGCAATTTGGACTATGATGATGATCAACCTGGTTCGAGTGGATGCAAGCAAATTGAGCCACTACTAGCTAGCTCAATGGAAGGTGGGTGGCCCAATATATACATACTCTCAATAGTCTTCGAAAGCAGCAGCCAGCTTGCTTGGTGATCTTTCACCAATATTCTTTTCGCCGGAAGGATGCATGCTGCTGGTGGTACAGAACAAAGGAGACAAGGACTCCAACTCCTGCCTTTTCTCCTACGGCGGCGGGGCTGGCCGGGCAAAAATGCAGCGTGTAAACTGCAAGATCAAGTCCTTCCATCCTAGTGAAGCCTTTTCCATGTTTTCACTTAAATGCCACCATTGATAAGACATGATATGATATTAAAAAAAAAAAAAAACACAAATCACATAAGCACATTCCATTAAAAGGAGGGTCATCAATCTCGAATATACCAAAAACTCAAAATCCTCGTCCCCAAAGAAACACAACAGTTCCTCCATTGTTAAAAAGTAATCAAAAGTGTAGCAAATGGGCTTATTCAAAGTCGCGGCTTTCCTATGGAGCCCTGAGTCCCATCATATTAGAACAAACAAACTACAATGCCAAAAATAGCCACCCATTTAAGAGAGGCATGAGTACGCCAAACTCTTCTCCTCACTCAGCTCCTCTGCAGTAGCATCCATCTTCTTCCTTGAAAATTCATCAATTGGAAGACCTTGAACAATGGTCCATTCTCCCTTTTTACATGCTACTGGAAAGGAATACATCAGTCCAGCAGGTACATTGTATGATCCATCAGAGTACACCCCCATGGAAACCCAGGTTCCCTCTGGAGTATCAAGAACCCAATCACGGATGTGATCACAAGCAGAACTAGCAGCAGATAAAGCACTTGAAAGTTTCCTAGCTTTGATAATTGCAGCACCACGTTGTTGGACAGTAGTTATGAACTCTTCGTTCAGCCATGCGTCATCTTTGACAAGCTCGCGGACAGGCTTGTCGCCAGATGGAGTTTTGACAGTTGCATGGTTGACATCAGGGTACTGAGTTGATGAATGATTCCCCCATATGATCACATTCTTTACATCAGAGACTGGAACGTTTAATCTCTCTGAAATCTGACCTAGTGCCCTGTTATGATCCAGTCTTGTCAAGCATGTGATATTCTTTGCAGGGATAGATGGTGCAAACTCCTTAAGGATCAATGCATTGGTGTTGGCAGGGTTGGCAACAACCAAAACCTTGCAATTGGGAGCAGCGTGCTGTTCCAGGGCAGCAGCCTGGGACTTGTAAATAGACACATTCTTTGACATCACATCTTTCCTCTCCATGCCTTCTTTTCTTGGGAAACCACCAACCATTACTGCAATATTGACTCCAGTGCATGCTTCAACAGCATCAGTTGTCGCAACAACGCCTTTAAGAAGAGGGAATGCAGCATCCACCAACTCCATTTTCACTCCATTCAATGCCTCAGCTGCAGGTGGAATATCGAGCATGTGCAAGATCACAGGCTGGTCAGCGCCCAGCATGATTCCCCGAGCAATCATGGGGACAAGAGCATATCCAATTTGTCCTGCGGCTCCAGTAACAAGAACGCGAACTGGATCTTTCGCCATTGATGAAGATCCGAGAACAGATCGAAGAGTTGATGAAGAACGAAGAGTGAGCTCTGGTACTACGATGCTGCGAACGATGGAGTTGTGAAGGAAATGAGACGACCAAGGCTCATTTA

# >c70757.graph_c0, MG516526

GAGGTCTCGAGACAGAATGGGGCGAATAGATCAGCGCCGCATTGCCTGCTTCTACTGTTTTCTCTTCACATTCTGTCTCGAGACCTCGTCTCTCTTTCTTATCTCTTTTCAAGCAAACTTCAGTTTCAGGATCAGCTGTCTTTGGATCCCTCCTCCGACTGATAGGTCGTGGTATCTTTTTCTTTTGGTGATTCGCTTTCATACTTGTTCTTCAGAAACCAGTAAAGAAAGCTTTGCAACTATGGCGGTCGCATTCTACGATCTCAGCTCTGCCGCTGGTCTGAAGAAGCTTGATGACTATCTGCTTACTCGCAGCTATATCACTGGATACCAAGCTTCAAAGGATGATATCACAGTTTTTTCTGCTATTTCCAAGTCTCCTGCTGAATATGTGAATGTATCCCGGTGGTACAAGCATATCGATGCACTACTGAGGATTTCCGGTGTTACTGGGGAAGGCAGTGGTGTGATTGTTGAGGGATCTGCTCCTATCACAGGAGAGGCTGTTGCAACACCACCAGTGGAGGACTCTAAGGCTGTTGCAGCTGCTGTGGATGACGACGATGATGATGTGGATTTATTTGGTGAAGAGACTGAGGAGGAGAAAAAGGCTGCAGAAGAGCGTGCTGCAGCTGTCAAAGCTTCTTCCAAAAAGAAAGAATCTGGGAAGTCATCAGTTTTGATGGATGTGAAGCCATGGGATGATGAGACAGACATGAAGAAGCTAGAGGAAGCTGTGCGAAGTGTTCAAATGGAAGGTCTCTTCTGGGGGGCATCAAAACTTGTCCCTGTTGGATACGGTATCAAGAAATTGCAGATCATGCTAACAATAGTGGATGACTTGGTCTCGGTTGACCATCTAATTGAAGAATATCTGACAGTCGAACCGATTAATGAATATGTTCAGAGTTGCGATATTGTCGCCTTCAACAAAATATGACCTGCTGAGCTAGAGTAGCAGTGAACTCTCCCCAGTAGCTTTTTGTCGTGTCGGCATTTCATTCTCCACTGCTATGGTGTGTGATTCTGTAGTGTTGCTTATTCCGTTTTCTTTCTTTAACATTCTGCTTTTGTACGGCGTACTGAATGTTCAAATCAATAACCTTTAGAAACTCTTTATCGATTAAGTTCTTTTATGCCCTGTTTGGGAAATACCTTTTGTTTCAATTAGCTGTATTGTTTTGGATAATTTTTTAATGAGTGGAATAGCTAACAACTAAACTTAAAAATAAAAAGTTACGGTTGATAGCTTTTTCAAAAATGCATTGAAAAAACTGTTGCCAAGGC

**>c67520.graph_c0**

TGAGTATATTATATTGGTCTCCTCCACCTCCTCCTCCTACTCCTCCGCTGCTTGCCATCTCCCAAAAGCCTAGCCTTCTTCTCGTCTCTTCTCTAGGGTTTTTGCGACCGAGGCCCTCTCCTCCCTCCCATGGCTTCTTCTTCTTCGTCTTCAGCTCATGCCATTAGATCCGTCGCCTGTTTCCGTTCGGACCTATCGCCGCCGTCCTTCAAGGCGTCGTCGAGCCTTGGTTTCAGCCGTAATGTCAACTTCTCCAAGACTCCCTCAACTTCGCTCTCTTCACCTTTCCAGCAATGCAGTTTCAGAAGTTTTCAACCCATCAGAGCCACAGCTACAGAAGTGCCACCAACAATTCAGAAACCACAAACAAGCGGGAAGACAAGAATCGGAATCAATGGTTTCGGCCGCATTGGAAGGCTGGTTTTACGTGTTGCAACATCAAGGGATGATATTGATGTCGTGGCAGTCAATGATCCTTTCATTGATGCCAAGTACATGGCTTACATGTTTAAGTATGATTCAACGCATGGAGTCTTCAATGGGACTCTCAGGGTTCTGGATGAATCCACCTTGGAAATCAACGGGAAGCAAATTAAAGTTGTGAGCAAAAGAGACCCAGCAGAGATTCCATGGGGTGATTTAGGAGCCGAGTTTGTGGTCGAATCATCTGGTGTTTTCACAACATTGGATAAGGCCTCAGCACATATCAAGGGTGGTGCAAAGAAGGTTGTTATTTCAGCTCCTTCTGCTGATGCACCGATGTTTGTGGTAGGAGTAAATGAGCAGTCTTATAAACCAAATATGGACATCGTTTCTAATGCAAGCTGCACTACAAATTGTCTTGCTCCTCTAGCCAAGGTGGTTCATGAGGAATTTGGCATTATTGAGGGGTTGATGACAACTGTGCATGCAACTACAGCCACACAGAAGACTGTTGATGGTCCTTCAATGAAAGATTGGCGAGGTGGGCGTGGAGCTGGACAAAATATAATCCCTAGCTCTACTGGTGCTGCAAAGGCTGTTGGCAAAGTCCTTCCTGAACTTAATGGAAAGCTTACTGGAATGGCATTTCGAGTACCCACACCTAACGTTTCTGTTGTTGATTTAACTTGTCGACTTGAGAAGAGTGCATCTTATGAAGATGTCAAAGCTGCAATCAAGTATGCTGCAGAGGGGCCACTTATGGGCATTCTTGGGTATACGGATGAGGATGTTGTCTCTAATGACTTTGTTGGGGACTCGAGGTTAAAAATTTCTCTCCCTTGCCTCTTTGGCATGCACATTCTTAAACATGCACGTTTCTTAATTGAGATGAACTGTAATATCTGGTGCTCACAGCTTGTGTGAATGTTGTCAACCCACCTTGCAAGGATCAAGGTTGAGGGCTTTGACCTATCATGAGCCTGCCATCCCCTTCTTTTTAATATGAGGGAGTGCGAGGATTTATCTTTTCTTTTTAATATGCTCTGATTGAGTGCCATACTCGCCATGAAATGGTGGATATGCTGTTTGCAATTTAGAAATTTTCTTGGACAAAACCAAGAGAACCTTAGGCTAAGATGTTTGAGAGAAAGATGTCAGATGATAATTTGTTTCCAGAGTTTAGTCGGTGTGATTTGATCTGACACTTTCTTGTCTCATCAGGTCAAGCATATTTGATGCGAAGGCTGGAATTGGGTTGAATAACTCCCTCATGAAACTCGTGTCCTGGTATGACAATGAATGGGGC

**>c74212.graph_c0**

ATTAAATACCTTCCACTTGCACTCTTCTTCTTCCCAAAAAGCTGCTCTCATCTTTCTATCCAAACCTTGCTGCTGCTCGTTATCATTGCCAATCGCCATGGCTTCGCAGAAGAAGATCAAGATCGGAATCAATGGTTTCGGAAGAATCGGTCGCTTGGTTGCCAGAGTTGCTCTGCAGAGGGACGATGTTGAGCTCGTCGCTGTCAACGATCCCTTCATCACCACTGATTACATGACCTACATGTTCAAGTACGACAGCGTCCACGGCCAGTGGAAGCACCACGAATTGAAGGTCAAGGACTCTAAGACTCTTCTCTTTGGTGAGAAGCCGGTCGCCGTTTTCGGATTCCGCAACCCAGAAGAGATTCCTTGGGGTGAGACAGGTGCTGATTTCGTTGTTGAATCTACTGGAGTTTTCACTGACAAGGATAAGGCAGCTGCTCACTTGAAGGGTGGTGCTAAGAAGGTTGTCATCTCAGCACCCAGCAAGGATGCTCCCATGTTTGTTGTGGGTGTCAATGAGAAGGAATACAAGCCTGAATTGGACATTGTTTCCAATGCTAGCTGCACCACCAACTGTCTTGCTCCATTGGCCAAGGTTATTAATGATAAGTTTGGAATTGTCGAGGGTCTCATGACCACTGTCCACTCAATCACTGCCACACAGAAGACAGTAGATGGACCTTCAGCAAAGGATTGGAGAGGTGGTAGAGCTGCTTCCTTCAACATCATCCCTAGCAGCACTGGCGCTGCCAAGGCTGTTGGAAAAGTTCTGCCTTCTTTGAATGGCAAGTTGACTGGAATGTCTTTCCGTGTTCCGACTGTGGATGTCTCAGTTGTTGACCTCACTGTCAGGCTTGAGAAGGCAGCATCTTATGATGACATTAAAGCTGCCATCAAGGAAGCATCCGAAGGCGATCTCAAGGGAATTATGGGTTACACCGATGAGGATGTTGTGTCTACCGACTTTGTTGGGGATAGCAGGTCAAGCATCTTCGATGCCAAGGCTGGCATTGCTCTAAGTCCCAACTTTGTGAAACTGGTCTCCTGGTATGACAATGAATGGGGTTACAGTTCTCGTGTGATCGACTTGATTGTCCACATGGCCTCTTGCTAGTCTGCATCATGCTATAAGCGTTGCCTGGGTTGGTTTCAAGGATTTCATGCTGGTTTAGTGTTTGAGGCTTAGACATGGTTACAGTACCAGAGTTACTGGATTGAAAATAAAAGCTTGTGGATTGCTTGTTTTGCAAGTTTTCTTATGTTTTCCATTTCATTTTTGGAGTAGTCAGTTCAGAGGGTTTATAGCTTTCCGAACTTTTTACCATTTTCAATTTCAGAAATAGAGATTGTGGTCATATTGCAGTTACTGTCCTTCCCAGTGTTATCAAATTGTGATTATTTTGTCACACTTCTCATTTCGGAAATCAATTTAGTTTAGTAATGCAAATCAATTTGGTTCTAGAAAATAAGAAGGAAATGTCTTCAAGACTTCAAGAAAACAACTTTCAGTGATTAATTTGGTTATAAATGGGCATATGTACGGCTGGCTATTTACAACCAAAAGCCAATCCTTCTAATTACAACCTTACCCATTTAAGAACATGAAGTACAAGCTCAAGGGTGGCTTCAAAGGGAAAGGGTCCCTTGTCCAGTACAACACCTGAACCCTGTGATTCTCAAGGGGTGACCCGGGAAAATGCAACAGAAGGACACAACGCCCCCGATCCAAGATTTTTGTTCCAATGAAATACTCCCTCTCTGTCCCAAAGTACTATCAACACATCAATAAAAAAAAAGTTTAAAATGAAATGATATTTTTATATTATTTTAGTCCACTTTTACCATTGGAAATGCAC

# >c70711.graph_c1, MG516529

CAAAGAAGTGGGGGGTGGCGGAATGCTAGTGGGCCCTAAACTTGAGATCATGGACGGGAGGTAAGCTTGTCTTGTCCAATAAAGCAGAAAGCGGCAATTTGGACTATGATGATGATCAACCTGGTTCGAGTGGATGCAAGCAAATTGAGCCACTACTAGCTAGCTCAATGGAAGGTGGGTGGCCCAATATATACATACTCTCAATAGTCTTCGAAAGCAGCAGCCAGCTTGCTTGGTGATCTTTCACCAATATTCTTTTCGCCGGAAGGATGCATGCTGCTGGTGGTACAGAACAAAGGAGACAAGGACTCCAACTCCTGCCTTTTCTCCTACGGCGGCGGGGCTGGCCGGGCAAAAATGCAGCGTGTAAACTGCAAGATCAAGTCCTTCCATCCTAGTGAAGCCTTTTCCATGTTTTCACTTAAATGCCACCATTGATAAGACATGATATGATATTAAAAAAAAAAAAAAACACAAATCACATAAGCACATTCCATTAAAAGGAGGGTCATCAATCTCGAATATACCAAAAACTCAAAATCCTCGTCCCCAAAGAAACACAACAGTTCCTCCATTGTTAAAAAGTAATCAAAAGTGTAGCAAATGGGCTTATTCAAAGTCGCGGCTTTCCTATGGAGCCCTGAGTCCCATCATATTAGAACAAACAAACTACAATGCCAAAAATAGCCACCCATTTAAGAGAGGCATGAGTACGCCAAACTCTTCTCCTCACTCAGCTCCTCTGCAGTAGCATCCATCTTCTTCCTTGAAAATTCATCAATTGGAAGACCTTGAACAATGGTCCATTCTCCCTTTTTACATGCTACTGGAAAGGAATACATCAGTCCAGCAGGTACATTGTATGATCCATCAGAGTACACCCCCATGGAAACCCAGGTTCCCTCTGGAGTATCAAGAACCCAATCACGGATGTGATCACAAGCAGAACTAGCAGCAGATAAAGCACTTGAAAGTTTCCTAGCTTTGATAATTGCAGCACCACGTTGTTGGACAGTAGTTATGAACTCTTCGTTCAGCCATGCGTCATCTTTGACAAGCTCGCGGACAGGCTTGTCGCCAGATGGAGTTTTGACAGTTGCATGGTTGACATCAGGGTACTGAGTTGATGAATGATTCCCCCATATGATCACATTCTTTACATCAGAGACTGGAACGTTTAATCTCTCTGAAATCTGACCTAGTGCCCTGTTATGATCCAGTCTTGTCAAGCATGTGATATTCTTTGCAGGGATAGATGGTGCAAACTCCTTAAGGATCAATGCATTGGTGTTGGCAGGGTTGGCAACAACCAAAACCTTGCAATTGGGAGCAGCGTGCTGTTCCAGGGCAGCAGCCTGGGACTTGTAAATAGACACATTCTTTGACATCACATCTTTCCTCTCCATGCCTTCTTTTCTTGGGAAACCACCAACCATTACTGCAATATTGACTCCAGTGCATGCTTCAACAGCATCAGTTGTCGCAACAACGCCTTTAAGAAGAGGGAATGCAGCATCCACCAACTCCATTTTCACTCCATTCAATGCCTCAGCTGCAGGTGGAATATCGAGCATGTGCAAGATCACAGGCTGGTCAGCGCCCAGCATGATTCCCCGAGCAATCATGGGGACAAGAGCATATCCAATTTGTCCTGCGGCTCCAGTAACAAGAACGCGAACTGGATCTTTCGCCATTGATGAAGATCCGAGAACAGATCGAAGAGTTGATGAAGAACGAAGAGTGAGCTCTGGTACTACGATGCTGCGAACGATGGAGTTGTGAAGGAAATGAGACGACCAAGGCTCATTTA

# >c72957.graph_c1, MG516530

AACTCCGACCCGAACCGAATATGCAACCAGCAGCAGAATCCGATTGAGCAGAAGCGCCAGCGGCGACTGGTAGCTGAGCCAAGCACCGCAATCATGAATCAATTCCTGAAATTCAGACCTTCGAAGAAAAGATTCGTCCCACAAGAAGAATCCTGATCTTACCAAAGTCCTACTCCACACTATCACTTGATCGCGAAATGACCTCCAATTCGGGCTCTTCTTCTTCGTCATCCTCCTCCGAAGATGAATCGGCCGGTAGGTGTTCTAGTACTGTTGCTAATTCGGCTCCTAAGCCTTCAAATGAACAATTTCGGTCTCTGGAACTGACGGAGGACGAAGAAGAATCGACGAGGGATTGCCTGGAGAATGGCGATGTGGCGATGAATGGGTTGTTCGATCCTGAGAATGAAGGTAGCCGAGTGGAGATTGTGGATGAGGCGATGAATGAGTTTGTTGATAGCGAGGTGGGATTTGATGGCGCTGGAGTTGTATGGGGGCGGACGAATTCGGAGCTTGAAGTGGAGCGGCCTACCAGCCCGGGCAGCAGCGGTTACGCCGGTGAGAGAGGGAGTAGCAGCGCCACGAGCGTGTCAAGGGCGGACGATGAGATCGAGGAAGTGACGAAGAACAATGGTGGCGTGGCGGTCGACGCTGTCCCCGATTCTCCTTCCGTCTCGTGGGTTCCGGGAAAACGCTATGGCGATGAGGATGATGCGTCTATATCATGGAGAAAGAGGAAAAAACACTTTTTCATTTTGAGTAATTCTGGGAAACCGATATATTCCAGATATGGAGATGAACACAAGTTGGCTGGCTTTTCAGCAACTCTACAGGCCATCATTTCTTTTGTGGAGAATGGAGGGGATCGTGTCAAGTTGGTGCGGGCAGGAAAGCACCAGGTGGTGTTTCTTGTAAAAGGACCAATTTATCTAGTATGCATCAGCTGCTCAGATGAACCATATGAATCATTGAAGGGACAATTAGAGCTTATCTATGGTCAGATGATTCTTATTCTAACGAAGTCCATTAATAGATGCTTTGAAAAGAATTCTAAATTCGATATGACACCACTTCTAGGAGGAACAGATGTTGTCTTCTCATCACTTATACACTCCTTTAGTTGGAATCCGGCCACTTTTTTGCATGCATACACTTGCCTTCCTCTTCCTTATGCTACAAGACAAGCTGCAGGTGCTATATTGCAAGATATTGCAGATTCAGGTGTACTTTTTGCAATTCTGATGTGTAAACACAAGGTTGTCAGCCTTGTCGGTGCTCAAAAAGCTTCCCTTCATCCAGATGACATGCTGCTGCTTTCAAACTTTGTTATGTCATCTGAATCATTTAGGACATCTGAATCTTTCTCTCCAATTTGCCTGCCAAGATACAATCCCATGGCTTTTTTATATGCGTATGTCCATTACCTGGATGTTGATACTTATTTGATGTTGTTGACAACTAGCTCGGATGCTTTTTATCATCTTAAAGATTGCAGGGGATTCGGATTGAATCAGTCCTCTTGAAGTCAAATGTTCTTAGTGAAGTTCAAAGATCCCAGATAGATGGTGGCATGAGGGTTGAGGACCTACCTGTTGACTCATTGCCTCAGTCTGGGTCTTCATCTCGTTTAGGGCAGCACAAACTTCAAACAGAGTCTCATGAGAGATCAAGAGAACCATTTGTTGGCATTGGTGGCCAGGCAGGACTTTGGCATTTTATTTATCGTAGCATATACCTGGATCAGTATGTGTCATCTGAGTTTTCTCCTCCAATCACTAGTCACCAACAACAGAAGAGACTGTATAGAGCGTACCAGAAGCTTTACACTTCAATGCATGATACAGGAATCGGGCCGCACAAAACACAGTTTAGAAGAGATGAAAACTATGTCTTGCTGTGCTGGGTTACGCAAGATTTTGAATTCTATGCTGCATTTGATCCTCTTGCAGATAAGGCATCTGCTATAAAGACGTGCAACCGTGTGTGCCAATGGGTTAAAGATGTGGAAAATGAGATATTTTTGCAGGGAGCAAGCCCCTTCTCATGGTGACATGACATGTATGTATGTCTGTCCAATTCTGTAGAACAGCAATGTAGTACTAGTGTATAACAACATAGGTCTTTGGTACTCGGTTTTCTGAAAACTCGACCCGTTCGCTTCATTTTATGGTATAGAGCAGCCGCAGCAGCAGTAGTAGTAATGTCTCTCCTGTTGGTGTTCATGTGAATCAATCATATGGCTAATAAATGGCATAATGAGCCACCCACCGGAGCGTATGTTGAATCCGGACCGGAGCCAATAAGTGAGTGGCCCTCATTTCTTAGGGATGAACTTGTGTTGTTGGTCCTTAGTAATACTAGCATCTCATGGATGATGTATACCAATATTAGTGTCTTAGGAAGTTTTCTTTTTATAAAATTAAAAAATAAAAAAATCTTAATACTCTCTCG

# >c60567.graph_c0, MG516531

AGCGACTGTAACGAAGTCATTTTGAAATACAATCATTCTGTAAGATAACAAATGTTTCGTGCAGCTCCTTTCTTCCACCCTGCTGTTGAATGGAACCTGATGACTTTTAATGCAAGAAGTACTACTGGAGCCCAAAACAATCATTTTAGTATTATTACACGACCCAAGCATTCTCCCTAAAATCAACCCACTTCATCAAAATGCTTACAGGAAGGACGATCCAATTTTTTCAAAGAAAAGAAAAACAGGTAGAATTACAGTGGTTCAGAAATGAAACAAAAGAGCACTAACAACACTATGCAGCAGAGAAGGTCTGTCAACAAACAAGTAGCACCTTTTAGTAGTCTTCTCCTTCATCCTCATCATCAGCGCCTTCTGCACCCACTTCCTCATAGTCTTTCTCCAGAGCAGCGAGATCTTCTCGCGCTTCTGAGAATTCGCCTTCCTCCATGCCTTCACCAACGTACCAGTGAACAAAAGCCCGTTTCGAATACATGAGATCGAATTTATGGTCAATCCGGCTGAAGACCTCAGCCACAGCTGTGTTGTTGCTGATCATGCAAACTGCACGCTGCACCTTTGCAAGATCACCTCCAGGTACCACTGTTGGTGGCTGGTAATTGATACCGCACTTGAAACCAGTTGGGCACCAGTCGACAAACTGAACCGTCCGCTTTGTCTTGATTGTTGCAACGGCAGCATTGACATCCTTGGGTACAACATCTCCACGATACATTAAGCAACAGGCCATGTACTTTCCATGCCTTGGATCGCATTTTGCCATCATGCTCGAGGGTTCAAAAACTGCACTCGTGATCTCTGGAACGGACAGCTGCTCATGGTAAGCCTTCGCAGCGGAGATTACAGGGGCATAGGATGAGAGCATGAAATGGATACGTGGGTATGGAACAAGGTTGGTTTGAAATTCAGTGACATCAACATTTATGGCACCATCAAACCTCAAGGAAGTGGTAAGAGAAGAAATGATCTGGGATATTAAGCGGTTCAAATTGGTATAAGTTGGCCTTTCGATGTCTAAGGATCTTCTGCAGATGTCATAGATAGCTTCATTGTCCAACAAGACCGCTACATCTGTGTGCTCTAGAAGGGAATGAGTTGACAATACGCTGTTGTACGGTTCCACAACTGCAGTTGAAACCTGAGGAGAGGGATAGATTGTAAATCCGAGCTTCGATTTCTTCCCATAATCTACAGAAAGACGTTCCAAGAGTAATGAGCCCAAACCAGAACCAGTACCACCACCAACAGCATTAAAAACCAAGAACCCTTGCAAGCCGGTGCAGTTGTCAGCTAATTTCCTGACTCTATCAAGGCAAAGATCAACGATTTCCCTTCCAACAGTGTAATGGCCTCTTGCAAAGTTATTAGCGGCATCCTCCTTGCCAGATATAAGTTGCTCAGGATGGAAGAGTTGCCTGTAACTTCCAGTTCTAACTTCATCGATGACAGTGGGCTCAAGATCAACGAATATAGCTCTCGGCACATGCTTTCCGGAACCAGTCTCACTGAAGAATGTGTTGAAAGCATCATGCGCAACACCTGGAGTTGTATCACTAGGCATCCTGCCATCAGGCTGGATTCCATGCTCGAGGCAGTAAAGTTCCCAGCAGGAGTTGCCCACCTGGATCCCAGCTTGTCCGATGTGAATGCTGATGATCTCCCTCATCTTCTCTACACACCAAAAGATGAGAGCTTTTTCTTCCGCCGCGAAAACTCGAAAGTGCCAACGTTGATGGGGTTAAAGAACGGGCTCAGGCAATCAAGCAAGCAAGCAAGAAGCTCGTTTTATTTTATGTTTCTTTTTCTTTTGTTTTGTTTTTCTTTTTTCAAATCTGG

# >c65147.graph_c0, MG516532

CGGCAATAACTAAATCGAGCCATTTTTTTCAAGTAGTGGCAAAAAGGTACCAATAGCGAAACGTTAATGGCAAATGCAAAACATTTTCCAAACATTCTAACAAAAGAGGAAAGATGAAACAAAACATGAACCACATTTCTCAGTCATAGGAGGATTATAAAACCTCTTTTGTTAGCAAGGAAAGCTTGAAGATGACACGCAGAATATGAAGTGAGAACAACCTCCCTTAAACATTCTAACAAAAGAGGGAAAGATGAAACAAAACATGAATCGATCATAAAAGCTCCCAATTCAATCAGGTATGAATCGACCAACGCACACAAACAACAAACACAAGAAAGCGACAAATAAGAGAAAGGATCTGATTACTCAGTATTCAGAACCCTCATCGCCTTCATCGTCAGCACCCTCAGCGCCAACCTCTTCATAATCCTTCTCAAGGGCAGCAAGATCCTCCCTGGCCTCAGAAAACTCCCCTTCCTCCATTCCTTCACCAACATACCAGTGCACAAAGGCACGCTTGGCATACATCAGATCAAACTTGTGGTCAATCCGGGAGAACACTTCTGCAACACTGGTTGAGTTTGAGATCATGCAGACAGCCCTCTGCACCTTAGCAAGGTCACCTCCTGGGACAACAGTTGGTGCCTGATAATTGATACCACACTTGAATCCAGTTGGGCACCAATCCACAAATTGGATTGTGCGCTTGGTCTTGATAGTAGCAACAGCTGCATTAACATCCTTTGGGACAACATCACCACGGTACATAAGGCAGCAAGCCATGTATTTTCCATGACGAGGATCACATTTCGCCATCATGGATGAGGGCTCAAAAGCACTGTTCGTGATCTCAGAGACTGATAACTGCTCGTGGTAAGCCTTCTCAGCCGAGATGACAGGAGCATAAGAGGAAAGCATGAAGTGGATCCTTGGGTAGGGAACCAGGTTTGTCTGGAACTCGGTCACATCAACATTCAGGGCACCATCAAATCTCAAAGAGGTGGTGAGAGATGAGATGACCTGAGAGACTAGGCGATTAAGGTTGGTGTATGTTGGACGCTCAATGTCCAAGGAACGCCTACAGATGTCATAAATTGCCTCATTGTCCAGAAGCACTGATACATCAGTGTGCTCAAGGAGGGAGTGGGTTGAAAGCACACTGTTGTAAGGCTCAACAACAGAGGTAGAGACTTGAGGTGAGGGGTAGATTGTGAACCCGAGCTTGGATTTCTTCCCATAATCAACAGAGAGACGCTCCAAGAGAAGTGATCCAAGACCAGAGCCTGTGCCACCTCCTACAGCATTGAATACAAGGAAACCTTGCAAGCCGGTGCAGTTATCAGCTAGCTTACGGATGCGATCCAAGCAGAGATCAACAATCTCCTTCCCAATTGTGTAGTGCCCACGAGCAAAGTTGTTGGCAGCATCTTCCTTGCCACTGATGAGTTGCTCAGGATGGAAGAGCTGGCGATATGTGCCAGTTCTTACTTCATCAATGACAGTGGGCTCGAGATCAACGAAGACGGCACGGGGGACGTGCTTCCCAGCACCTGTTTCACTGAAAAAGGTGTTGAAAGCATCATCGCCACCACCGACAGTAGCACCAGACAACTGGCCATCAGGCTGCAATCCATGCTCGAGACAGTAAAGCTCCCAGCAGGCATTTCCAACCTGAATACCAGCCTGACCAATGTGAATAGAAATGCATTCTCTCATCTTCGGAATAAAAAAAACAGCAAAGAAGAAGCGGAAAAAAAAACCCTAGAATTTGGGGGAAAATGCAAAGGGCGTTTATGAAGACGCCTTTCAGAGAAGTGAGGTTAAGATTTGAG

# >c57696.graph_c0, MG516533

GTGTGTCAATTATATTGTGCACTTGTTTGTCCGCACTTGCGCACTTTGTGCGAAACTATGTGCGGACTGTTGTGCGTAGCGCACAACTTCAGGTGAATAACAAAGGACTACTCACGTGAACAACTAAATTCACTTTGTTCCTATTCACTTCAGATCAGTGTAGAAAACGGTGAATCACGCGGTCCTACTCAGGTGAAGGAGGAGGAGCGAAAAAATCAGTTATTCACCCGAAAAAACTCAAGTTTGAAACAATAATAATACATAGTGAAGTGAAATGGTAGAAGAAGTGATAAATCAGTCAGCTCTGAAGCTCAATAATTGCTACAGCTGCTTCAATACCTAACTAGTACTCTACTAATTACCTAAAACCCTCTTCTTATTCTTCAGCAAGCCTTATTCTTTACTCATCTTTGCATTTCACTACATTCGGCTGCAAAGAAAGAATAAGCACCACCTAATAAACTTCAGTCTTCCATGCAATCCGTACATAAACAGACTTGCAACACCTAATAAACTTCACCCCTTTTAACTACAACACCATACAAGAGGGTTGCGCGTGCATTCTCTCTCACTAATTCTCTAACTTTCCATTACACATACCGCTGATCGGATTCACCGTTATCTCCTTGTGGATGTAAATCACTCCTTTATCTCCATCCACAACCTGCCCACTTCCTCTAAGAATCCATCTCGTGGTCAGCAATCCCTCAACTCCAACAGGACCCCGGGCATGGATTCTACTTGTACTAATCCCAACCTCTGCACCCAGTCCAAACCGGGCACCATCACAAAACCTCGTGCTTGCATTATGAAAAACAGCAGCACTGTCAACTCGACTTAGAAATACGTCAGCCACTACACTGTCCTCTGTGACAACGCAATCTGTATGGGCACTTCCATATTTGTGAATATGATCAATGGCAGCGTAAACATCATCAACTATTTCAACTGTGCATGCAGTTGAATTGTACTCGTGTTCAAAAGAATGTGCCTCTGGAATATTTAGTACGGCACTCGCCCTTGGTCCACCGTATAAAGTCACACCTTCAGCACGAAGAGCAAAAACAATTTCATTAAGACCACCCGTCTTTGTGAAATCCTTATGCACAAGGATTGTCTCCATGGCATTACAAGCTGCTGGATAATCCAATTTAGCATCCAAAACAATCCGTTTTGCCTTTTCCACATTAGCAGACTTGTCCACAAATACATGACATATTCCGTCAGAATGGCCCAACACAGGAATTTTCGTTGAACTCTTGATCGTTGAAACCAGTTTATTGCTGCCCCTTGGGATGACTAGATCAATCACGTCATCCAGCTTGAGGAGATCAGGGATCTCATCTCTTGAAGTCACTAGTCCAATAAGCTTCCTTCCAACATTTTCTGGAATGGCTTCAGTTATGACCTTATGTAAGATAGCATTAGATCGCATTGCTTCTTTTCCTCCTTTCAGAAGAAGGCCATTCCCACTTCGGATTGCTAATGAGGCAATCTGTCGAATTCAAATATAAGTAGATGAAGCCACTCAAAAGCTAAGAACAATATATTTAAATGTAGACATAAAAAGCGCCTAAGTTCACATCAGAAAATAATTCATTTGAATATGTCTAATTTTCATTTGAACACTTCCAACTCCATGATATAAGCTACAAATAGGCAAAAGACGTAAGATCAAAAACACTATTAGAAAGAATATACTGATTGAACTTAAACATGATCTGATACATTTATATGGTTTCAAAAGAATTGGAAAGCTATTTGCGTTATCATTTTTTTTTAGCAAAAATTACTATTGTATTAGTTGGATCTGAATGTAACATAGGCTGAAGATATTATAGTCCATTCCAAATAAGAAAAATGGACACTTTACCCCTCGAAGGTAATTAATAGAGAGACTCAAGTTTTACATCTTTATGATTAGTGTGAACATATCAGCCAAAGGTAATGCTGGGAGAATTTCAGAGCTAATCTAAGTTGGAAAAGTAGAAATCAAAGAAGCTAAGAGAACATGATTAAATTGTAAAACAAGCTAAGTCTATTACATAAAAACCAAGATACCGGTGTCAGGGGGGGAAGTGAGAACAAAATCAAATTATACAAGTTATGGCTTTAAGGAATGAAGATTTTCTGATTCCAAACATTATTTTGTTCATATGTAAAGAACATTATCATATGTAAAGATTTGTCCAAATTCTGTGAAGCAGTTTAACCTATAGTAGTACAACATTATTTTGTTCAGTTTTTTTAGCAATACCAATGTTATTGCCTTAATCAGATAGAGTAATTAGTATTTTGTACTCCGTATAATGATTGTGGCTATATGACCAAGTTTACTGCTTCATCAAAGCAAGGTCGGTGTCGCTGAGAATACAATACCAGCATACAGAACATAATAATGCCTTTCACACTATCAAAAACAATACCAAAAGAGTAATCTGAAAGTTAGAGATTATAGTCCACCTGCACTAATGCATCAGGCCGCGCCTCAAAAACAATTAAAAGGACACCTAGTGGGCATGATGTTTTCTCTAAAAGGAGTCCTTCTGCAAGCTAGGAGGAAACAATAAAATATATCTAAATCAAATAGTTAGTTGCAGATATTTCAAATAAATAAATGACACTAAATTATCCTAAAAAGCAGCTTCTTTTCTAACAATTTATAGTGTCTAAAAAATGCTTCATACCTCAAACTGAAACCTAAATTACTTAGGCAATCATACTTTCAGGACCTAAAACTTCAGAAAATACCAGCAAGACCATAATACCTGTGTTTTCTTCAGAACACGGCCAATTGGATCATCCATATTTGCAAGTACACGAACTGAATTTGCAAGGCTTCGAATCTTCCCAGGCTTCAGAGCCAAACGAGATATCAAGGATTTTTCGTATCCAGCTTCTTGTGCAGCAGCAACATCAGCTTCATTTTCTGCAAGGATCGATTTTTCATTTGCTACGAGGGCATCAGCTATGTCCAATAAAATCCTCTCTCTTTCTTCTGAAGACAATCCCTGAAGCCTCCTAGAGCTATCTCTCGCACCAATTGCCATTTCACGTGCACCAACTTCCCTAATTGGGTTCCAGAACTGTGCATCCCGATGAAAGAGAGTACCAACTCGTTGCCCTTGAAGAACTTTGATTATGTTCCCAGCTGCAAACCCACTGGTGATAACAACAGGAATACCAGCAGATGCTGCACTTACAGCAGCTTTCACTTTTGCAGTCATGCCCCCTCTCCCCACCCTCGATTTGTCACCGAAAGTGATTTCACTTTGATGTTTTTCCTTAATATATGTGTGAATTAGCTTTGATTTTGGATCACTGGGGGGACCACTGTAAAGACCGTCAACATCACTCAATAAAACAAGAAGATCAGCATTTAGTTCCAGTGCCAATAGAGCTGCCAAACTGTCATTGTCCCAAAATATACCAGAAGAGTCCTCATAAGGAGCTTTCCTAGTGCTGACTGCATCGTTTTCATTGAATATTGGAATAACCTTCAACTCCAATAATGATTTGACAGTTTCACTTAGTTGTTTTCTGAAATCTTTGTCTCGGAAATCACTGTCCGTCACAAGAAGTTGAGCTGATGACATATCCAGCTGCAGACAAAGAAGAAAAGATAAGAGACAACTTTTAAATGTACCCCATACATAAACCTGGTCTTGACATGGACAAACTGGGTGTCATCCTATAAGTAATATAAAGTAGGTGGGGAGGAAAAAAAACTTCCCCGTGTAGCAGAAAAGGAAGATGAAACTAAGACCTGACCAAACAATGTATCATATAGAGCCAAGAGACTGTTCTGTCCAACAGCAGCACATGCCTTCCCATGAAGTTCTCCTTGCGGCTTCTGAAGATCAGAAATGCTGCTATTTACTAATCTCCTGTATCTCAGCCTTTGGCGACCAAGGCCAACAGCCCCAGAGGTAACCAAAATAACCTCATATTCTTGAGTAATTAGCTCTTTTATCTGTTCACATAGTGCTCCGAGTCTTCCTAGAGCTAATCGTCCATCATTTCTAGTGACAACAGCTGTTCCAACCTTGATAACAAGACGCTTGACATCCTTGACAAAAACACGAGTGGAATCCATGTCGTCCATACCCGGAATTGCGGAGCGAGTTGCCGGTGACGCAGACTCGGTGGTGAGTCGCCGCGTCGGGATGTGAAGCAGAAGCAGTATAAGATTGAATGATAGGGTTTGTTTTTTTTTTTTTTCTTTTTTGTTTCTTCCACTTAGCACTTCATTTTGACATCTGAGGTTGGCCATGCATACAAGAGCAGCAGCCTT

# >c73625.graph_c0, MG516534

TAGTTCATTTGATACATCTCTAAACAGAAAATGAGGCAATTTGTTGGGAATAGCAAATTGATTGGCTTGAGTATCATTTAGCTGATTGGACTCTATGTAGTTGGATCACATGAATGATCTTGGTACATAGCGGATGATTTGAGAAACATGAAGCGAGCCGTAAAAGTTGATACTTTGAAAAATCGAAAGAGAGAAGTTTTGGAAAAGTCACGAGCTTACCGTCCAACAGGAAGCATGGAAATCGTGCACAGGTGGCGCGTGGGGACTTGAGCAAGATCCACCATCCAAAGTTTTTAACTTTTTTCTGTTCTTCTTCTTCTTCCCTCCCTGTTTCCTCGGAACGCCTTGATCGTTCTCTGTTTGGTGTTTTTTGATCGTGCTGATCATGGCTTCCGTTTTCTCTTCTGCACCCAGTTACGCCCTCCCGCCGATTTTCTCCGGCGTTAACTCTTGCTCGTTTCATGCCCGACCGCCGGGTTCGATCTCCGTGCAATCATCACCGTCTCTGTCACTTCCGAAACGACGTCGTGTTCATTTCGCTGCCGGAGGAGCCACCGTCAACTCTGTTCCCGCACGAAATGGGGTGGTTTATACAGTTGGTGATTTCATGACCAGAAAAGAAGATTTACATGTTGTGAAAACCTGGACTCCTGTTGATGAAGCATGGGAGGCTCTTGTCCAGAAGGGAGTAACTGGTTTCCCTGTGGTAGATGAAGATTGGAACTTGGTTGGTGTTGTTTCTGATTATGACTTGCTAGCTCTTGATTCCATATCAGGCACAAGTGAAAGTCAGACAAGCGTGTTCCCGGATGTCAATAGTTCTTGGAGAAGATTCAATGAAATACAGAAACTGGTTAGCAAATCTGATGGGAAAGTTGTTGGTGACTTGATGTCTCCTGCTCCACTTGTTGTCCGTGAAATGACAAACTTGGAAGATGCTGCTAGGTTGCTGCTGGAGACAAAATTCCGGAGGCTTCCCGTGGTAGATGGTAATGGCAAACTTGTTGGAATCATAACAAGAGGAAATGTTGTGAGGGCTGCCCTAGAGATTAAACGGTATGCCGAGCAGCATATGTGACTCCTGGTCATGGACTTGCAGAAGATGAATTTTCAGTTTGTTCAAATTCCTGCAGTTGTTTGTCTTTGGTTAGGGCAGTGCTTTTGCTCTGCATAGATCAGTTGTAGAGAGTTAATTGGTACAACAGTCAGGAGAATGTTTGTGTATTGGTCAGTGACCATCAACACAAGCTTAGAACATTTAAAAGTTCTGTATATTTGATACTCCATTAGAAGGATGTATATGATAGTAGTAGATATTTGTTAACATGCATTACGATTGTCAGTCTTTCAACTTCCTGTTCTCATTAGTTTCAACTTTTGCTTTTTGTTTATCTTTTTTGGTTCATTTAGCTAAATCTTCTTGATGGTGAAGATCCGGTATGCATCGTGATTTGTGCACATGTCTAACAGATTCACACCAACATACTTTTATGGCAGATTAGGTTTCAAGAATTTCAAGTTCAAAATGTTAGCTAGCAATGAGTTCCACCATAGCAAGCTGAGAATGCCAAGCCATTACAGTAAGCTGGTGATGTGAATTTTCCAGTGCAAATGTATCAAATGCCATTAATATTCTAAAAGATTGCACATGATATGTAGTTCCATTTCATATAATACCTACATGTTTTGATTTACTTGGTTGATGCAGTTTTGATCTTGTTTGCAGACAATTCTTGAATGAGAGCATTGACATCAGAGTGAGATAAGCCTCCTCCCTCTTGTATGACTTTTCTGGCCATATCTGCATATGATTTAGCCTTGCTTCTCATCTCATCAACTCCATTACCTCTCATCAGCTCTGTAACAGCTTTCTGTATTACATCCCTCTTCACTAAATCCCCAGTTGGATGATTCCACTTCTTTGCACCCACACTAATCCCAATCCGCAGAACCTCGGTGACTAGCTTCTCGTTGTAGAACTGCTCAGCTCCGACTGGCCATGTGACCATGGGAACCCCATCGCAGACTGCTTCAAGGACAGTTCCAACCACAATGTGTCACAAACCCTCCATTTGCTTGGTGCCTAAGGATTAATGATTGGGGTGCCCATCCTCGAATGATGAGTCCCTTGCCGCGCACTCTTCCCTCGAATCCTTCTGGTAACCATTCCTCTTTCCCTTCTTTCTTGTCCTTCACCACCCAAATGAATGGCAGATCTGAAGCTTCAAGACCCATTGCGATTTCGAAAAGCTGGGAAGAATCAAAGTCAGCCATACTCCCGAAGCACATATACAACACTGATAACTGATTTCAGTTGCTTGGTACGCAGCCATTCGAAGCACTTGTCCTCCGGTTCTCCATCATCATCATCATCATCCCATACCACAAAACAAAGGCTCATGTATGTTGGCCCTAATATTTACTGTTACATCCCCATCCAGTGAGATTACCTCCCAAGCCAAGCCTTCACATTTGAGGAGTCAGGAACATCTGAGGAGCATCACATCTTTTCCCAGAAGAGTAACCAGGTCGGTTTCTATAGATTGGTACCTGAAAAAGAATACGTACGGTGGTACACAATAATCCGGATAGAGAGTCGTATGTTATGTCGATATCGTACACCCTAATTCCTTTGCAGCAATGTCAAGAAACTGAATCTGCTTTTCGCCTGCAAGTTGGCTATGAGCTTCCCATGTCAAGCACTTCTCTATATGAGCTTTCCAATCTACATCTACGTTGAAATATCGGTAGGAAGGGTCATGGATGCCATTTCCATTTATAAGCACTTCTTTAGAGCTATACTTGGGTGAACCTGTATGCAAGATGTCACGCAGAACTGATATGCTCAGAGCACGAACATGTGCGCTCGGATGAGTGAGACACCGGATAGTAGGTGGTAGCCGACACTTAAGAAGGTTTGAAAGACCATCTGCAACCGCCAGTCCAGACTCTCCAAACTCCAGTATAGGTGATACTGCTCTTGCTGTTGCTTCTAGTAGCTCTAGTTGGGGTAAGGTGCAAGCTTCTCCATCCACAAGAACTCCGTCTGTGGCACGCAGGAGTAGATCTGATGCACTAGCTACAATTACCAAGCATTCTGGGCTATCATGATTTCTCATAAGTTCCACAATCAACTTCACAATCCGCTGGTTAACTTTCCACATTTTCTGATTTTGTTCATCATCCTTTGCAATCCACGGCTGCAATTCCCTCTCCGCCTGAAAAACCACTGCTGTAGCGGCTTTTGTTGGAGATGCCAACACCACATTGCACAGAGCATCAACCACCTGTCTCCATCCTTGCTGAGCAGATGTGCTCTCTGCAGTAGGCTGTGTTTCAGGCGCTGCCATCAGTTTGTGCCATAATAATGAAACCACAGAAAAGCACAGTTCTTGTTTCTCAACAAGTATCGATCTCAGAAGAACCTGGGCACTGCAATTATATCCTAGGTGTCTGTCCATAGTGAGGAAATTGGCTAAATCAGAAGCATCTATTGGGAAACTTTCAACTCTTTTACCCGAGGAACTTCCTGATCCTTCATCTGGAGGCAATGCATTCTTGCATCTATGATTAGCATCTGAAAGGACTGAATCCTTACATTCTGAAGTAGAAGGTTGTCCAACATCAAAGCTACTAGTTTTTGTGCATTTATGCTTCCTTGTATCCAAACCATTGGATACATTCTTCCGAATATTTGCATGGACTAACCGTAATTCTAATGGTTCAGCCTTGTTAACAATAGATGCAACAGCCTTGCTATGAATGTCGATAAGATTGTACAATGATGATGCTCTGGCAAAAATTTCACTATCCCACTTGCAATGCACAAATACGGAGAGCGCCTGCATACAAGACTTCGAACGTCTAAAGAGTTCAGAAACGTGAGCTGCAACCATAGCTGCTGCAACAATTTCATTTGAACTGTAACTCCATGAAGTACCGACAGAAGATGGCTTGAGAGAGAATAGTGCCTCCAAAATTGCTAAAATCCTGTGTGTATGATGAATTGCTGAGTTAATTCCGTTTCTGTAATCATTACATTGCCCATTAACTTTTGCAGGCTTTGCTGGAGTTCGTGCACCACTGGGGACACAATGGTTATCCTGCCTTGAAATTATAGGAAATATCTGGAGCTCACATGCAAGAGCACAAACAGCAGCTAGAACATAAGAATCAAAAGCTGCCACTGGCCCTTGTTTCTTCCCCTTCCTGAATTTCAAGTTTTTCGGCTTTTCGGATGTCCCCTGAAATTCTTCGATGCTTTCATTTTGGGAGAAAACTTCCTCATCCCGTACTCTCTTGCTCCTAATTGACTGAGCTTCATAGCTCACACAGACAGTTAAGACAACAAAAAGTAGGCGTGAAGCAAGCTCTATCGAGGCACATGATTCTACAAAAAGAGAATGGACCATCGTACGGAGCTCAGCCACAGCAAGATTCTTAGAAGCAGAGCCAATGCTGTATCCAGTTTTTCGCATCTGCTCTCGGGAAGACTCAGGTGGAAAGGTTCTCTGGAGAATTGCTTCCACAGTAGCCACAAAGATTTTCATCAAACATGCTTCAGAGGGACTACCACGTGGAAGATATTCCAGGACTTTGAGGAAAGGTATGTAGAGATTCCATGACAAGATGGGAGGTTGTGTCGGAGTTGAGACAATGATTTCAGGAAGATCAACTGCTGAAGAGCTTAAAGGTATAAGGCCATATGCAGCTTCCCAGATTGTACAGATTCTCCATTCAACCTCAGGCCCGTGAGCACAAAGCATGGACGCAATCCCTTGGGCAGTGGCTTCAATAGTTGCTTCAGCTGCAGCAACTTCTACCTGCTTCCTGTAAGATGAAATATAGCCACCAGAAGATTCATGTTGAACTTCAAAACCCTCAATTTGCCTCAATGGAGGAAACAGCAAAGCAGGCTGCGACAGTATGCGAAAAAGCAAAGCTGCTGCAGCATCAGCAGCTATCCCCACTCTCATTGACATTGCAATCCCAATTGCACGCAGAAAATGTAGATGCATCCAGTTTGGTGGAAGCCTTATGCCTGAAGCATAGTCTTCAGCAGCATGAAGGAGTTCAACTAGCTGTACAGCAGCATCAAGTGCATCAGGTGCCCACGATGGTGGCGCTTCTAGAAGTCCTACAAGAAGTCTCTGTGTAGCACTTGGAGTAGCAATGGCATAGTACCGATGAAATAAACGTGCATAAGGCTCAAGAGCTGGCAGTCCTGCTACTAAATGCTCATCCAAAGCTGTTGTTGGGGGAGGAAGGAGAAGGGCAGGGACTGCTACAGCTGTTAAAGTAGCAGTCTCATAACGAGCAACCTCATCATCACATACACTCAATATTACACCAGCACCATTCGCGACTGCCCACCTTGGAGTGGATGGCATAAGCTGAGGATGCTTCCCAGATCCACGAGAAGAAGCAGTTGATGGTGGCTTAAGTTCTCCTGCTGCGTATTTGCCCATGACTCCACTGCACCAGCGGAAGTAGTCACTTCTAATACCCAAGGGTGCAGCAAGCAATATATCAGTGATCCAAGGGGACAAAGGTCTCAAAGGTTTCCTTTCCTGCTGCATAAAGGGTGCATCACAGGATGATTTACCATTTACAGAATAACTGGTTGTAGCACAGCTGCTGGTGGCACTTCTATCTGTTTCACTATTTAGCGGTTCCACCTTATATACTGGACGATTGTAATGTGTCAAAATACGCAATATCTCGCCACATGCCAGAGCCCACTGTTCAGAGTACTCGGTCTCACTACTTGGGCAGACCAATGAAATGAAAGAAGCAAATGGAGCATTGCTCTTATCATATACAAGTGTACCATCAATGATACATGAAATAATAGGGAGAACAACAGCATGCCCATGCTCTGGATGGTGAAGGACAAAAGTCGCCAGAACATCATCAAATAGGCGCTTTTCATTTGACGGATAACCGCTTCGAATCAGCTCAGCAATATCATCTGGGAATGTTTCTGAAGTAAATTGCCCCAAGTACTCCACATATGCAGTAACTTGAGCCTTTCGCTGTTGATGGTCTAGAGGCGGAGGCCAGAATAACAAGGAGGACTGAAGTCCGTCAATCCACCTCTCGGATGTGCCAGCCATTATAAGTAAGAAATGTGTACACTAAACAGAAAAGCTATTTACTTTCTGCGCTTATGAAGAAACAAGGAAACAGAGCAGCATGCCAATCCACAACCTAGAGCCTGACGTTCAACAATCAAGAATCAGTATTTCGTAGTTCAACATCCCAAACAAGCCAGCCGAAAATCCCACGAACCACAAGCTACGAAACACGTAATAGAGCTTAAATTGGTGTAGAATATCATAAGTTACAGCAAACTCTCGCAAGAACGGAATATGGTAAATCCCAACACTGTACTGTTCTTAAAAGCTCTCCGCTTTGGCCATTCTCGACCTCTAAAGCCTGACCAAAGCGAACCTGGACATGGAGAGAGAGAATTGCATGGATCAGCAGCTTGTCTGGTTAGATTAACAAAGACAAGACTTGAACAAACAGATTGAGCTGTGTTTTCTAAACTGTTTAATCCTAATTTCTTTTGCTGGGAAATCTTTTCCAAGGTGGAGAGTGAAAGAGGATCTTTAGAAGGATCGTTGTTCTTTGAGAGAGTTGCAGACGTGGCGATGATATTTTCCTCTACAATCTTCTTCGTTTCCCTTGAGAACTTTTATTCTAAAATCTTTTCCTCTTGCTGCTGAGAGAACTAAAATCTTTTCCTGTGTGGAGAGTGGAAAGAGGATCTTTAGAAGGCCTGTTGTTCATTCAAAGAGTTGCAGACGTGGCGATGAT
